# Supplementary material for: HEXIM1/P–TEFb complex controls RNA polymerase II pause release and immediate early gene induction following neuronal depolarization
Source: J Biol Chem. 2026 Feb 25;302(4):111325. doi: 10.1016/j.jbc.2026.111325 (PMC13049930; doi:10.1016/j.jbc.2026.111325)
Supplement: SuppTable 2 [file mmc3.docx]

| **Gene and primer direction –**  **forward (f) or reverse (r)** | **Sequence (5’ to 3’)** |
| --- | --- |
| *Hprt*-f | GGAGTCCTGTTGATGTTGCCAGTA |
| *Hprt*-r | GGGACGCAGCAACTGACATTTCTA |
| *Egr1*-f | AGCGCCTTCAATCCTCAAG |
| *Egr1*-r | TTTGGCTGGGATAACTCGTC |
| *Egr1*-pre-mRNA-f | CATCGGGAGTCAGTGGTAGC |
| *Egr1*-pre-mRNA -r | AGGGAGAAGCGGCCAGTATA |
| *Egr1*-promoter-f | CCCACCACCCAACATCAGTT |
| *Egr1*-promoter-r | GGGTAGTTGTCCATGGTGGG |
| *Fos*-f | TACTACCATTCCCCAGCCGA |
| *Fos*-r | GCGCAAAAGTCCTGTGTGTT |
| *Fos*-pre-mRNA-f | GCAAATCACACTCTGCCTGC |
| *Fos*-pre-mRNA-r | GCTGCAGCCATCTTATTCCG |
| *Fos-*promoter-f | CGTAGAGTTGACGACAGAGC |
| *Fos-*promoter-r | TGGACTTCCTACGTCACTGG |
| *Hexim1*-f | CCTGATCTCAAAACCGGCCT |
| *Hexim1*-r | AATCCTCATCGCTGGTGTCG |
| *Arc*-f | ACGATCTGGCTTCCTCATTCTGCT |
| *Arc*-r | AGGTTCCCTCAGCATCTCTGCTTT |
| *Arc*-pre-mRNA-f | ACAGGAAGCAGCAAGATGGT |
| *Arc*-pre-mRNA-r | ACAGAGCCAGGAGAATGACAC |
| *Nr4a2*-f | AGCCACCTTGCTTGTACCAAATGC |
| *Nr4a2*-r | TTGTAGTAAACCGACCCGCTGTGT |
| *Nr4a2*-pre-mRNA-f | GTCTCCTGAAAACCTGCCCA |
| *Nr4a2*-pre-mRNA-r | AGCCCACGTCGATTCCAATC |
| *Gapdh*-f | AATGGTGAAGGTCGGTGTG |
| *Gapdh*-r | GTGGAGTCATACTGGAACATGTAG |
| *Rps10*-f | TCTCGAGGCTACGTGAAGGA |
| *Rps10*-r | TGGATGCCCTCGTTCGTAAG |
| *Vegfa*-f | TCTCCCAGATCGGTGACAGT |
| *Vegfa*-r | AAGGAATGTGTGGTGGGGAC |
| *Tcf4*-f | CGAATCACATGGGACAGATG |
| *Tcf4*-r | CTTGCGTCTGCGATTCATAA |
| *Hdac2-*intron12-f | AGCATGCCATTACAGCACCT |
| *Hdac2-*intron12-r | AGGCTACTGCCCACTCCTTA |

**Supplementary Table 2.** Primers used for RT-PCR and ChIP-qPCR analysis.
