# Supplementary material for: HEXIM1/P–TEFb complex controls RNA polymerase II pause release and immediate early gene induction following neuronal depolarization
Source: J Biol Chem. 2026 Feb 25;302(4):111325. doi: 10.1016/j.jbc.2026.111325 (PMC13049930; doi:10.1016/j.jbc.2026.111325)
Supplement: SuppTable 3 [file mmc4.docx]

| **Antigen** | **Company** | **Source** | **Dilution** | **Catalogue** | **Lot** | **Isotype** |
| --- | --- | --- | --- | --- | --- | --- |
| HEXIM1 | abcam | Rabbit | 1:1000 (WB/ICC)  6μg/IP (ChIP) | ab25388 1mg/mL | GR3245992-1 or  GR3460630-1 | IgG |
| Beta-ACTIN | Cell Signaling | Mouse | 1:2000 (WB) | 8H10D10 | 21 | IgG2b |
| CCNT1 | Cell Signaling | Rabbit | 1:1000 (WB)  3μg/IP (ChIP) | 81464 D1B6G | 1 | IgG |
| CDK9 | abcam | Rabbit monoclonal | 1:1000 (WB)  3μg/IP (ChIP) | ab239364 | 1023428-1 | IgG |
| FLAG (M2) | Sigma | Mouse | 1:1000 (WB) | F1804 |  | IgG1 |
| Tubulin, beta III (Tuj1) | Millipore | mouse | 1:2000 (ICC) | TU-20, MAB1637 | 3407183 | IgG1 |
| NeuN | Abcam | Mouse | 1:500 | ab104224 |  | IgG2b |
| GAPDH | Abcam | Mouse monoclonal | 1:2000 (WB) | ab8245 | 1035914-4 | IgG1 |
| Rabbit IgG | Thermofisher | Rabbit | NA | 02-6102 | SJ257848 | IgG |
| RNAP2-pS2 (3E10) | Millipore Sigma | Rat | 1:500 (WB) | 04-1571 |  | IgG1 |
| RNAP2-pS5 (3E8) | Millipore Sigma | Rat | 1:500 (WB) | 04-1572 |  | IgG2a |
| Total RNAP2 (F-12) | Santa Cruz Biotechnology | Mouse | 1:200 (WB) | sc-55492 |  | IgG2b |

WB = Western blot, ICC = Immunocytochemistry, ChIP = chromatin immunoprecipitation.

**Supplementary File 3. Antibodies used in this study.**
